# Habitat and tree species identity shape aboveground and belowground fungal communities in central European forests

**DOI:** 10.3389/fmicb.2023.1067906

**Published:** 2023-03-06

**Authors:** Benjamin Hofmann, Lukas Dreyling, Francesco Dal Grande, Jürgen Otte, Imke Schmitt

**Affiliations:** ^1^Institute of Ecology, Diversity and Evolution, Goethe University Frankfurt, Frankfurt, Germany; ^2^Senckenberg Biodiversity and Climate Research Centre (SBiK-F), Frankfurt, Germany; ^3^Department of Biology, University of Padova, Padua, Italy; ^4^National Biodiversity Future Center (NBFC), Palermo, Italy

**Keywords:** biodiversity, fungal diversity, metabarcoding, tree microbiome, rhytidome

## Abstract

**Introduction:**

Trees interact with fungi in mutualistic, saprotrophic, and pathogenic relationships. With their extensive aboveground and belowground structures, trees provide diverse habitats for fungi. Thus, tree species identity is an important driver of fungal community composition in forests.

**Methods:**

Here we investigate how forest habitat (bark surface vs. soil) and tree species identity (deciduous vs. coniferous) affect fungal communities in two Central European forests. We assess differences and interactions between fungal communities associated with bark surfaces and soil, in forest plots dominated either by *Fagus sylvatica*, *Picea abies*, or *Pinus sylvestris* in two study regions in southwestern and northeastern Germany.

**Results:**

ITS metabarcoding yielded 3,357 fungal amplicon sequence variants (ASVs) in the northern and 6,088 in the southern region. Overall, soil communities were 4.7 times more diverse than bark communities. Habitat type explained 48–69% of the variation in alpha diversity, while tree species identity explained >1–3%. NMDS ordinations showed that habitat type and host tree species structured the fungal communities. Overall, few fungal taxa were shared between habitats, or between tree species, but the shared taxa were highly abundant. Network analyses, based on co-occurrence patterns, indicate that aboveground and belowground communities form distinct subnetworks.

**Discussion:**

Our study suggests that habitat (bark versus soil) and tree species identity are important factors structuring fungal communities in temperate European forests. The aboveground (bark-associated) fungal community is currently poorly known, including a high proportion of reads assigned to “unknown Ascomycota” or “unknown Dothideomycetes.” The role of bark as a habitat and reservoir of unique fungal diversity in forests has been underestimated.

## 1. Introduction

Tree-fungus interactions are fundamental for the functioning of forest ecosystems. Fungi form mutualistic, pathogenic, and saprotrophic interactions with forest trees. For example, mycorrhizal fungi improve nutrient and water uptake by forest trees (Gerdemann, 1968), and endophytic fungi associated with leaves can promote tree growth (Doty, 2011), or prevent infection by pathogens (Herre et al., 2007). Pathogenic fungi cause severe diseases of forest trees, such as beech bark disease (Cale et al., 2017), while soilborne saprotrophic fungi are essential for litter decomposition, energy flow and nutrient cycling in forest ecosystems (Baldrian et al., 2012). Since trees are keystone species in forests – forming extensive above- and belowground structures – they provide ample and diverse habitats for fungal communities. Ecological niches provided by trees are, e.g., the phyllosphere, rhytidome (bark surface), and rhizosphere, including the soil surrounding the root. Fungi are able to occur in several of these habitats, connect and exploit them (Baldrian, 2017). To obtain a more complete picture of the interaction of trees with their fungal communities in forests, we need more information on fungal communities simultaneously associating with different tree niches (e.g., Yang et al., 2022a). This will help us understand the fungal linkages between above- and belowground habitats, or between living and dead tree tissues. One confounding factor to such analyses is that some ecological niches in forests, e.g., the bark surface, are little known, especially with respect to fungal diversity (Dreyling et al., 2022). The comparison between the bark surface – the largest, seasonally stable aboveground structure – and the soil is one step toward understanding the “ecosystem microbiome” (Baldrian, 2017) of a temperate forest.

The rhytidome, or “dermosphere” is defined as the outer bark surface of living trees (Lambais et al., 2014), and constitutes a terrestrial habitat with an estimated surface area of 0.9 ha/ha in woodlands (Baldrian, 2017). Despite its vastness, the bark micro-ecosystem of forests remains largely unknown with respect to fungal diversity. Bark provides mechanical and chemical defense to the tree, for example against herbivores and pathogens, and thus drives a process of selection with regards to fungi and other microbiota that that are able to exploit the habitat (Lambais et al., 2014). It consists of different biomolecules like starch, sugars and xylem exudates as well as cellulose, hemicellulose, lignin, and xylan (Martins et al., 2013). In general, the rhytidome is characterized by nutrient scarcity, since the described biomolecules cannot be easily exploited by microbes as carbon sources (Buck et al., 1998). Other characteristics of the bark surface habitat are desiccation, the secretion of mycotoxic substances like tannins or terpenes (Buck et al., 1998), or exposure to solar radiation (Baldrian, 2017). Nevertheless, tree bark provides microhabitats in form of cracks and cavities with favorable conditions for the survival of microorganisms and the attachment and germination of fungal spores (Buck et al., 1998; Magyar, 2008). Arrigoni et al. (2018) described the bark of fruit trees as a likely reservoir of potentially pathogenic or beneficial fungi and bacteria.

Tree species identity is known to be an important driver of tree-associated fungal communities. Forest trees actively recruit and select mycorrhizal fungi, resulting in species-specific ectomycorrhizal communities (e.g., Lang et al., 2011). Deadwood from different tree species features specific fungal communities (e.g., Krah et al., 2018; Yang et al., 2022b), with broadleaved dead trees generally exhibiting higher diversity compared to coniferous dead trees (Rajala et al., 2010; Purhonen et al., 2020). The type of litter produced by different tree species influences the community composition of soil fungi and bacteria (Urbanová et al., 2015) and the diversity of genes involved in lignin degradation (Barbi et al., 2016). Concerning fungi of the phyllosphere, tree species identity (Kembel and Mueller, 2014) and tree genotype (Bálint et al., 2013) shape community composition. In some cases, tree species identity is a stronger driver of fungal communities than abiotic or other environmental variables (Krah et al., 2018; Saitta et al., 2018).

In this work, we analyze diversity, composition, and structure of fungal communities associated with bark surfaces and soil in temperate Central European forests, focusing on three common tree species (*Fagus sylvatica*, *Picea abies*, *Pinus sylvestris*). Specifically, we address the following questions: 1. How does the dominant tree species in a forest plot influence bark-associated and soil-associated fungal communities? 2. What are the compositional differences between bark and soil fungal communities? 3. Which fungal taxa are shared between the aboveground and belowground communities?

## 2. Materials and methods

### 2.1. Study regions

We collected environmental samples of bark surface- and soil-associated fungi in two study regions in Germany, which are part of the Biodiversity Exploratories^1^: Swabian Alb in the southwest and Schorfheide-Chorin in the northeast. In each region 50 forest plots (100 m × 100 m) were selected, which represent typical Central European temperate forest vegetation of various management intensities (Fischer et al., 2010). Regional differences between the sampling regions are summarized in Table 1. The dominant (most abundant) tree species on a forest plot was determined based on a stand composition assessment of all trees with a diameter at breast height (DBH) greater than 7 cm (Schall and Ammer, 2018). The most frequent dominant tree species in both study regions was European beech (*Fagus sylvatica*), followed by Norway spruce (*Picea abies*) in Swabian Alb and Scots pine (*Pinus sylvestris*) in Schorfheide-Chorin (Schall and Ammer, 2018). Thus, we sampled beech and the dominant coniferous tree species, respectively, in each study region. Bark and soil samples were taken from the same 20 m × 20 m subplots, in a total of 94,100 m × 100 m plots, during the month of May in 2021. For each plot we generated one composite sample for soil (from 14 subsamples) and one composite sample for tree bark (from 6 subsamples).

**Table 1:** Differences between the study regions Swabian Alb and Schorfheide-Chorin (Fischer et al., 2010).

Study region	Swabian Alb	Schorfheide-Chorin
Area	422 km^2^	1,300 km^2^
Geology	Calcareous bedrock, karst	Young glacial landscape
Altitude a.s.l.	460–860 m	3–140 m
Annual mean temperature	6.0–7.0°C	8.0–8.5°C
Annual mean precipitation	700–1,000 mm	500–600 mm
# of sampled forest plots	50	44
Dominant deciduous tree species	*Fagus sylvatica* (37 plots)	*Fagus sylvatica* (28 plots)
Dominant coniferous tree species	*Picea abies* (13 plots)	*Pinus sylvestris* (16 plots)
Average (min / max) proportion of dominant tree species per plot	80% (43%/100%)	84% (47%/100%)

Dominant tree species are the most abundant species per plot based on a forest assessment (Schall and Ammer, 2018).

### 2.2. Sample collection

We obtained soil samples from the 2021 Biodiversity Exploratories Soil Sampling Campaign. In brief, 14 soil samples were collected per plot from seven equidistant sampling points along two 40 m transects. Soil was sampled with a split-tube auger (diameter 50 mm) from a soil horizon of 0–10 cm after the removal of twigs, litter, and the organic soil layer. The fourteen subsamples per plot were mixed and sieved to <2 mm particle size for Schorfheide-Chorin and < 4 mm particle size for Swabian Alb. For each plot, 5 g of the composite sample were stored at −20°C until further processing. Given this sampling design, it cannot be ruled out that the soil samples are influenced by plants other than the dominant tree species. However, the *Picea* and *Pinus* forests we sampled were nearly monocultures, and the *Fagus* forests did not feature extensive below-canopy vegetation. Furthermore, we sampled from the mineral horizon, so that potential influences from mosses and small herbaceous plants with shallow roots (e.g., geophytes) are less likely. Lastly, we included a large number of plots with the same dominant tree species, so that potential effects of other under-canopy species are expected to be leveled out.

To analyze the rhytidome, we collected six subsamples per plot from the trunk bark, 150 cm above ground, from six spatially random individuals of the dominant tree species. Following Dreyling et al. (2022), we selected two small trees (DBH 5–15 cm), two medium size trees (DBH 15–30 cm) and two large trees (DBH > 30 cm), to account for community variation between age groups. After mobilizing the biofilm *via* spray-moistening with sterile water, we used sterile nylon swabs (FLOQSwabs™, Copan, Brescia, Italy) to collect the sample. These swabs were moved around the trunk in a 3 cm wide band (Dreyling et al., 2022). The six swab tips of each plot were pooled in a 15 ml tube containing 5 ml of Nucleic Acid Preservation (NAP) buffer (Camacho-Sanchez et al., 2013), kept on ice in the field and subsequently stored at 4°C until DNA extraction. We took one blank sample per study region. For this we exposed six swabs to ambient air and processed them in the same fashion as the environmental samples.

### 2.3. DNA extraction

We extracted DNA from 150 mg of soil from the composite sample, using the Quick-DNA Fecal/Soil Microbe Microprep Kit (Zymo Research Europe GmbH, Freiburg, Germany) as specified in the manufacturer’s protocol. Cell structures were disrupted by mechanical bead beating for a duration of 6 min (SpeedMill PLUS, Analytik Jena, Jena, Germany). Bark samples were processed with the same extraction kit. However, an additional pre-processing is required to liberate the biological material from the swabs (Menke et al., 2017). Thus, each composite sample was diluted with 5 ml of ice-cold phosphate buffered saline (PBS), transferred to a 50 ml tube (to allow movement) and vortexed for 30 s (Menke et al., 2017). Afterward, 1.5 ml of the resulting suspension were transferred to a 1.5 ml tube and centrifuged at 6000 × g for 15 min (Menke et al., 2017). The supernatant was removed and beads and buffer from the extraction kit were directly added to the remaining sediment. Consecutive steps followed the same treatment as the soil samples. DNA quantity was checked *via* photometry (Implen NanoPhotometer™, Pearl Implen GmbH, München, Germany).

### 2.4. PCR amplification and high-throughput sequencing

The DNA extracted from the environmental samples was amplified with primer pairs targeting for the internal transcribed spacer 2 (ITS2) region. We selected the forward primer fITS 7 (GTGARTCATCGAATCTTTG) (Ihrmark et al., 2012) and the reverse primer ITS 4 (TCCTCCGCTTATTGATATGC) (White et al., 1990). Each sample was amplified in triplicate following a double index multiplexing scheme for each replicate, with individual octamer barcodes attached to both, forward and reverse primers. Each PCR run contained eight PCR negative controls (i.e., no sample added to the reaction) and the two extraction blanks, adding up to a total of 104 × 3 samples after PCR. To assess potential primer jump at sequencing (Schnell et al., 2015) during the bioinformatic processing, we included 23 empty wells as so called “Multiplex Controls.” Each reaction (total volume 15 μl) contained 5 ng of DNA, 7.5 μl hot-start polymerase (MyTaq™ HS Mix 2X, Meridian Bioscience Inc., Cincinnati, United States), 0.6 μl with 10 μM of each primer and 4.3 μl DNAse free water (Dreyling et al., 2022). The initial denaturation was executed at 95°C for 60 s, followed by 35 cycles of denaturation at 95°C for 15 s, annealing at 56°C for 15 s and extension at 72°C for 10 s, as well as one final extension step at 72°C for 60 s (Dreyling et al., 2022).

The PCR products were cleaned by magnetic bead purification (MagSi-NGSPREP Plus®, magtivio BV, Nuth, Netherlands) according to the manufacturer’s protocol. The final DNA concentration was determined by fluorometric measurement, using the Qubit dsDNA HS assay on a Qubit® 3.0 Fluorometer (both Thermo Fisher Scientific Inc., Waltham, Massachusetts, United States). Afterward, the cleaned PCR products were pooled equimolarly and sent to Fasteris SA (Plan-les-Ouates, Switzerland) for library preparation and sequencing. To avoid bias and chimera formation from additional PCR during the library creation, we chose the Fasteris MetaFast protocol. The amplicons were sequenced on an Illumina MiSeq (Illumina Inc., San Diego, California, United States) with 2 × 300 bp reads.

### 2.5. Bioinformatics

Reads were supplied by Fasteris SA with adapters removed [Trimmomatic (Bolger et al., 2014)]. We demultiplexed the sequencing reads using Cutadapt v3.3 (Martin, 2011) in two runs, once with barcodes in forward and once in reverse complement orientation, with the recommended settings for dual-indexes (error rate 0.15, zero indels allowed, minimum sequence length 50 bp). This is required due to the mixed orientation libraries obtained from the PCR-free library construction. Any remaining primer sequences were removed with Cutadapt v3.3.

Amplicon Sequence Variants (ASVs) were determined following the DADA2 pipeline for ITS amplicons (Callahan et al., 2016). The filtering and trimming was applied with default parameters (maxN = 0, truncQ = 2, minLen = 50, rm.phix = TRUE, compress = TRUE, multithread = TRUE), except for the number of expected errors maxEE, which was set to c(6,6) to take into account the mixed orientation of the sequencing library. Following the denoising and sample inference steps, read-pairs were merged within each triplicate and chimeras were removed from the dataset, resulting in one ASV table per triplicate. Because of the mixed orientation, we checked for reverse complement reads with DADA2s *rc*() function (Callahan et al., 2016) and added them to the original ASV table. In a last step, the three replicates were merged into a single table, which was matched against the UNITE database (version 9.0, including eukaryotic ITSs as outgroups; Abarenkov et al., 2022) by the DADA2 *assignTaxonomy*() function (Callahan et al., 2016) with minBoot = 50 and tryRC = TRUE. The ASV table was checked for potential contaminants with the decontam algorithm (Davis et al., 2018), set to both prevalence and frequency, and further curated using the LULU algorithm (Frøslev et al., 2017) with default parameters, grouping ASVs through patterns of similarity and sequence co-occurrence.

### 2.6. Data analysis

We analyzed diversity and composition of fungal ASVs in R (R Core Team, 2021) with the R Studio Environment (RStudio Team, 2021). Taxonomy tables, ASV tables and sample data were combined in phyloseq objects, which are specifically designed for the analysis of microbiome data (McMurdie and Holmes, 2013). Figures were generated in R with functions from the ggplot2 package (Wickham, 2016) and arranged using the ggpubr package (Kassambara, 2020), if not stated otherwise. Samples were not rarefied but viewed as count data (McMurdie and Holmes, 2014; Gloor et al., 2017). Prior to the community analyses, we split the dataset by study region to exclude any influence of variance in the regional conditions, such as climate, soil properties or elevation (Table 1). The number of samples per region and dominant tree species within the region can be found in Table 1.

#### 2.6.1. Alpha diversity

We visualized ASV richness in relation to the number of reads per sample as rarefaction curves, generated with the *ggrare*() function from the ranacapa (v0.1.0) package (Kandlikar et al., 2018). We calculated alpha diversity [Shannon Index (Shannon, 1948)] of fungal communities with the *estimate_richness*() function (McMurdie and Holmes, 2013). We determined whether fungi were specific to a particular habitat using the *ps_venn*() function of the MicEco package (Russel, 2021).

#### 2.6.2. Taxonomic community composition

To display the taxonomic community composition at order level, phyloseq objects were subset to the 25 relatively most abundant taxa with the *top_taxa()* function of the fantaxtic package (Teunisse, 2017). Barplots were generated using the function *plot_composition()* from the microbiome package (Lahti and Shetty, 2017). To explore influences of different habitats and host tree species, the plot for each study region was split into these two categories.

#### 2.6.3. Beta diversity

The effect of host tree species and habitat on fungal community composition was assessed with a non-metric multidimensional scaling approach (NMDS). To generate the NMDS, we used the function *ordinate*() set to “NMDS” and using Bray–Curtis dissimilarity. We visualized the result with *plot_ordination*() (McMurdie and Holmes, 2013). The variation between the groups included in the NMDS was reviewed with a Permutational Analysis of Variance (PERMANOVA) using the *adonis*() function (Stevens, 2019) from the vegan package (Oksanen et al., 2020). The variation within the groups was tested with the *betadisper*() function and verified with a permutation test with the function *permutest*() (Oksanen et al., 2020).

#### 2.6.4. Variance partitioning

To assess how much of the variation in the data was explained by habitat and tree species we used variance partitioning analyses for both alpha and beta diversity. To partition the variance for alpha diversity we used the *varPart()* function of the modEvA package (Barbosa et al., 2013) with three linear models, containing (I) both explanatory variables, (II) only habitat and (III) only tree species. For beta diversity the variance was assessed based on the ASV table using the function *varpart()* from the vegan package (Oksanen et al., 2020). This function uses redundancy analyses to assess the amount of variance explained by the individual fractions of each explanatory variable and their overlap. Both methods used adjusted R^2^ values (Table 2).

**Table 2:** Top five hub-taxa (ASVs) from co-occurrence networks.

ASV_ID	Phylum	Class	Order	Family	Genus	Species
(Swabian Alb)						
ASV_1676	Basidiomycota	Agaricomycetes	Agaricales	Inocybaceae	NA	NA
ASV_257	Ascomycota	Dothideomycetes	Capnodiales	Cladosporiacea	*Cladosporium*	NA
ASV_258	Ascomycota	Dothideomycetes	Pleosporales	Nigrogranaceae	*Nigrograna*	NA
ASV_28	Basidiomycota	Agaricomycetes	Phallales	Phallaceae	*Phallus*	*Impudicus*
ASV_790	Ascomycota	Dothideomycetes	Pleosporales	Cucurbitariaceae	*Neocucurbitaria*	*Quercina*
(Schorfheide-Chorin)						
ASV_1227	Ascomycota	Leotiomycetes	Helotiales	Leotiaceae	NA	NA
ASV_257	Ascomycota	Dothideomycetes	Capnodiales	Cladosporiacea	*Cladosporium*	NA
ASV_27	Ascomycota	Lecanoromycetes	Trapeliales	Phlyctidaceae	*Phlyctis*	*Argena*
ASV_28	Basidiomycota	Agaricomycetes	Phallales	Phallaceae	*Phallus*	*Impudicus*
ASV_839	Basidiomycota	Agaricomycetes	Thelephorales	Thelephoraceae	*Amaurodon*	NA

Hub-taxa were defined as taxa with the highest number of shortest connections to other ASVs (betweenness centrality).

#### 2.6.5. Co-occurrence networks

Co-occurrence networks of the fungal ASVs were generated for each study region with the *SPIEC-EASI* algorithm, which is based on co-occurrence and relative abundance (Kurtz et al., 2015). To reduce the number of nodes for better visibility, only ASVs that constitute more than 1 % of the reads were included. The applied parameters for the algorithm were lambda.min.ration = 0.01, nlambda = 70, pulsar.select = TRUE, seed = 10,010 on 50 subsamples (rep.num = 50), method = “mb” (Meinshausen and Bühlmann, 2006), and sel.criterion = “bstars” (Müller et al., 2016). The graphics for the networks were created in Gephi v0.9.2 (Bastian et al., 2009) with the Fruchterman-Reingold Layout (Fruchterman and Reingold, 1991) and considered modularity (Brandes, 2001) as well as betweenness centrality (Brandes, 2001; Blondel et al., 2008). The modules determined *via* Gephi v0.9.2 were assigned to different habitats by aligning the ASVs to the habitat types (i.e., tree species and above- / belowground).We calculated hub-taxa for each network, which we defined as the five nodes in the network with the highest betweenness centrality (i.e., the highest number of shortest paths; Freeman, 1977) with the *betweenness*() function from the igraph package (Csardi and Nepusz, 2006).

## 3. Results

### 3.1. Alpha diversity

**Figure 1:**
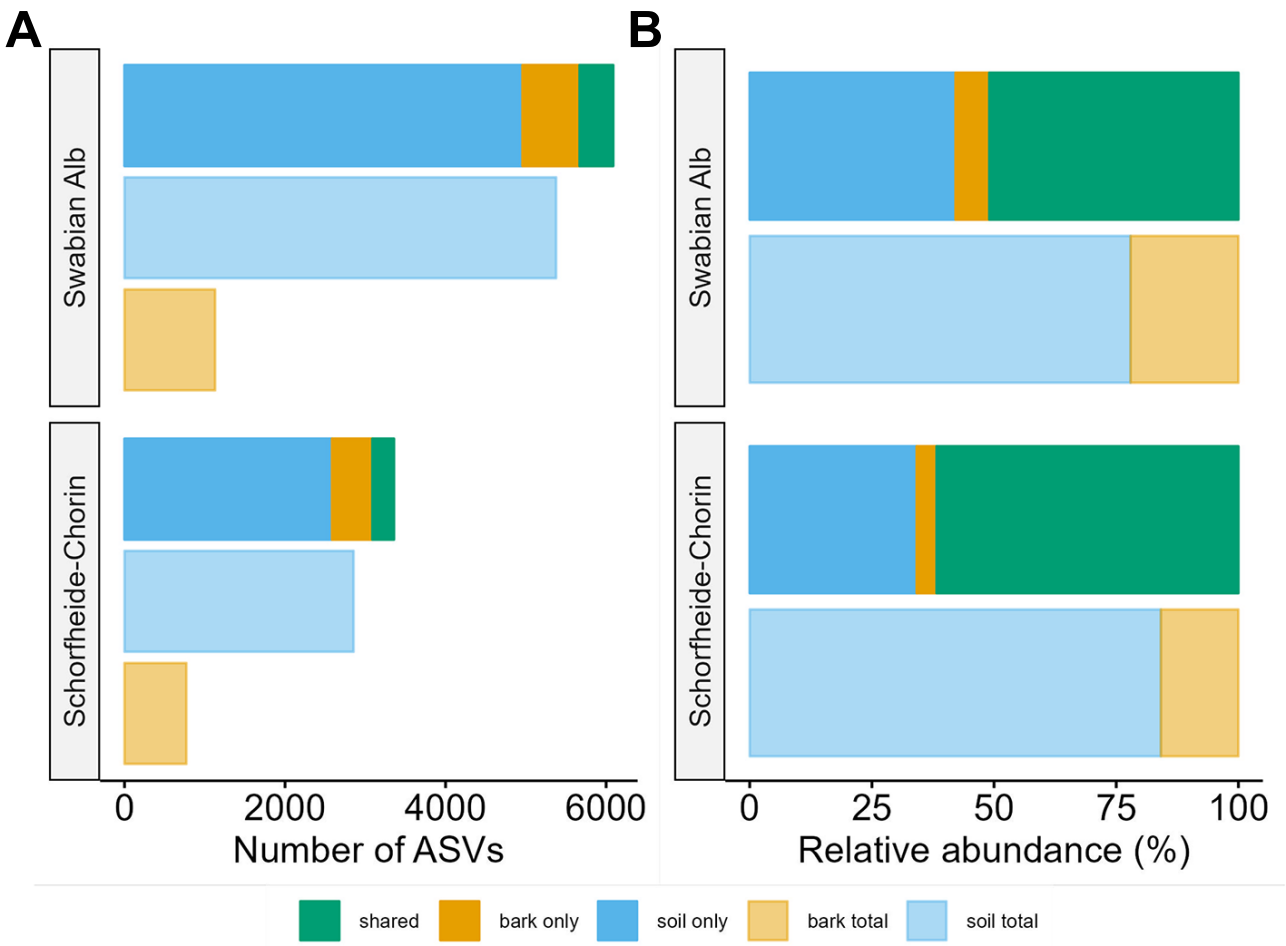
Fungal diversity associated with two forest habitats: bark surface and soil. Diversity is presented as total number of amplicon sequence variants (ASVs) **(A)**, and relative abundance of ASVs **(B)** in soil, on bark, and in both habitats. Data are presented for two regions, Swabian Alb and Schorfheide Chorin.

**Figure 2:**
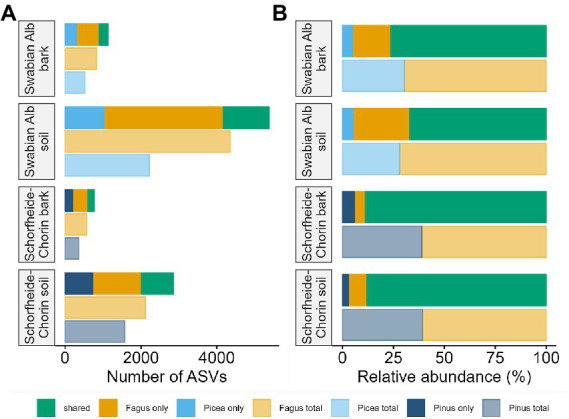
Fungal diversity associated with different tree species, and different habitats. Diversity is presented as total number of amplicon sequence variants (ASVs) **(A)**, and relative abundance of ASVs **(B)**. In each of the two study regions, Swabian Alb and Schorfheide Chorin, we compared fungal communities associated with a deciduous and a coniferous tree species.

The metabarcoding approach yielded an average of 39,245 reads for the Swabian Alb, and an average of 43,607 reads for Schorfheide-Chorin. The resulting number of ASVs in soil samples (7,033) was about 4.6 times higher than the number of ASVs in bark samples (1,497), suggesting a strong effect of habitat on fungal diversity. This effect is highlighted by a significant difference in Shannon diversity between the habitats within each region (Wilcoxon test: *p* < 0.001 for both). The habitat effect is also visible in the rarefaction curves (Supplementary material S1). The curves reach saturation plateaus, suggesting a low number of undiscovered ASVs and sufficient sequencing depth. Bark samples yielded fewer reads than soil samples, regardless of tree species identity and study region, while the read distribution bark/soil is similar in both study regions (Figure 1). The number of taxa shared between soil and bark surface was low (on average 343), but the abundance of these taxa was high (on average 62% of the reads; Figure 1 and Supplementary material S2). Considering each tree species, the number of associated fungal ASVs was generally higher in the Swabian Alb (*Picea abies* = 2,602, *Fagus sylvatica* = 4,872) than in Schorfheide-Chorin (*Pinus sylvestris* = 1,833, *Fagus sylvatica* = 2,499). Additionally, fungal diversity was significantly higher in the Swabian Alb (6,088 ASVs) than in Schorfheide-Chorin (3,357 ASVs; Wilcoxon test, Shannon diversity: *p* < 0.001), indicating an effect of geographic location. *Fagus sylvatica* had a higher number of associated fungal ASVs than the coniferous tree species in soil and on bark surfaces in each of the two study regions (Figure 2A). However, Shannon diversities between tree species within a study region were not significantly different (Wilcoxon test p < 0.001). The number of taxa shared between the coniferous and deciduous tree species was low (on average 1,018 ASVs in soil, and 195 ASVs on bark), but the relative abundance of these taxa was high (on average 78% of the reads in soil, and 83% on bark) (Figure 2B).

### 3.2. Beta diversity

**Figure 3:**
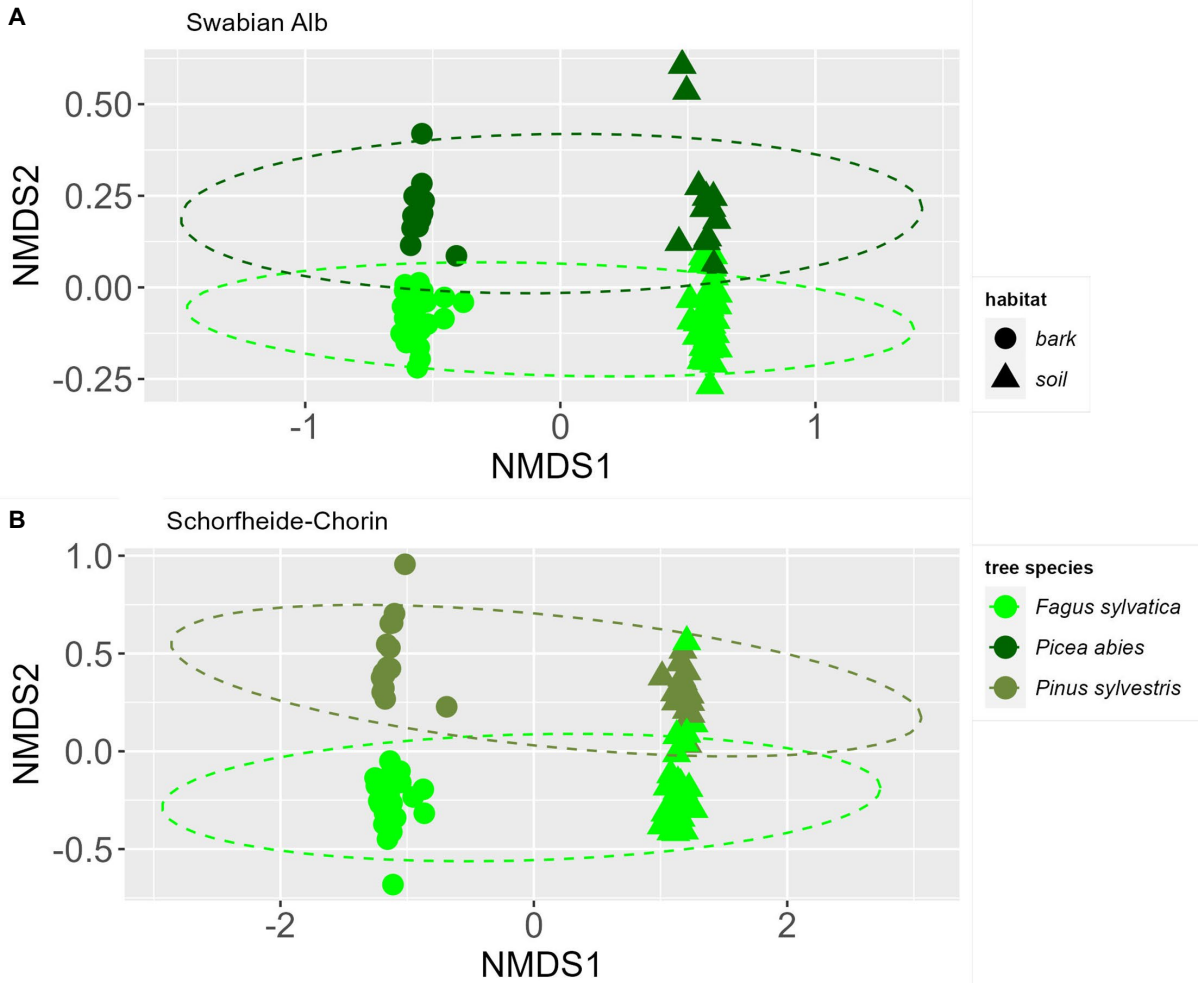
Similarities of fungal communities associated with bark or soil in forest plots dominated by different tree species. NMDS ordinations of fungal communities at two study sites: Swabian Alb **(A)** and Schorfheide-Chorin **(B)**. Ellipsoid hulls encircle the dominant tree species (indicated by colors). Symbols indicate the two habitats, bark and soil.

The NMDS ordinations indicate a strong effect of habitat (bark surface vs. soil) on the fungal community composition (Figure 3). In addition, the fungal community is affected by the dominant tree species identity. The communities show a significant difference between dominant tree species in Swabian Alb and Schorfheide-Chorin, which is confirmed by PERMANOVA tests (p < 0.001) for both study regions. Permutation tests of the homogeneous dispersion within the groups (by tree species) confirm that there is no difference in the inner group distribution of the samples (*p* > 0.05) for both study regions.

### 3.3. Taxonomic community composition

**Figure 4:**
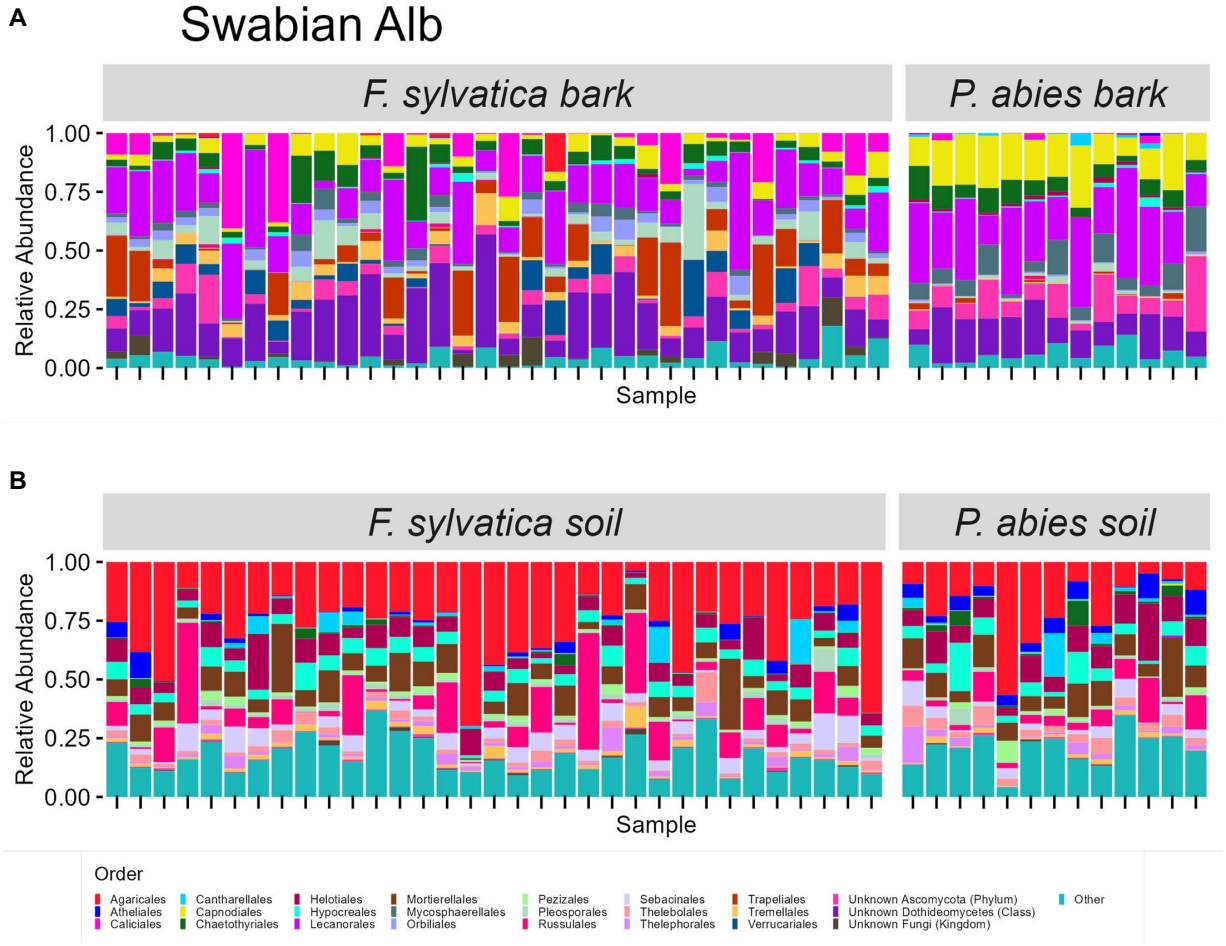
Taxonomic composition of fungal communities in Swabian Alb. **(A)** fungal groups associated with bark surfaces and **(B)** fungal diversity associated with soil. Taxonomic composition is shown at order level, faceted by dominant tree species. Each bar represents one sample (= one plot) and shows the relative abundances of fungal phyla. Only the 25 most abundant orders per plot are shown. The remaining orders are summarized as ‘other’. Some groups were not assignable at the level of order and are designated with higher taxonomic ranks.

**Figure 5:**
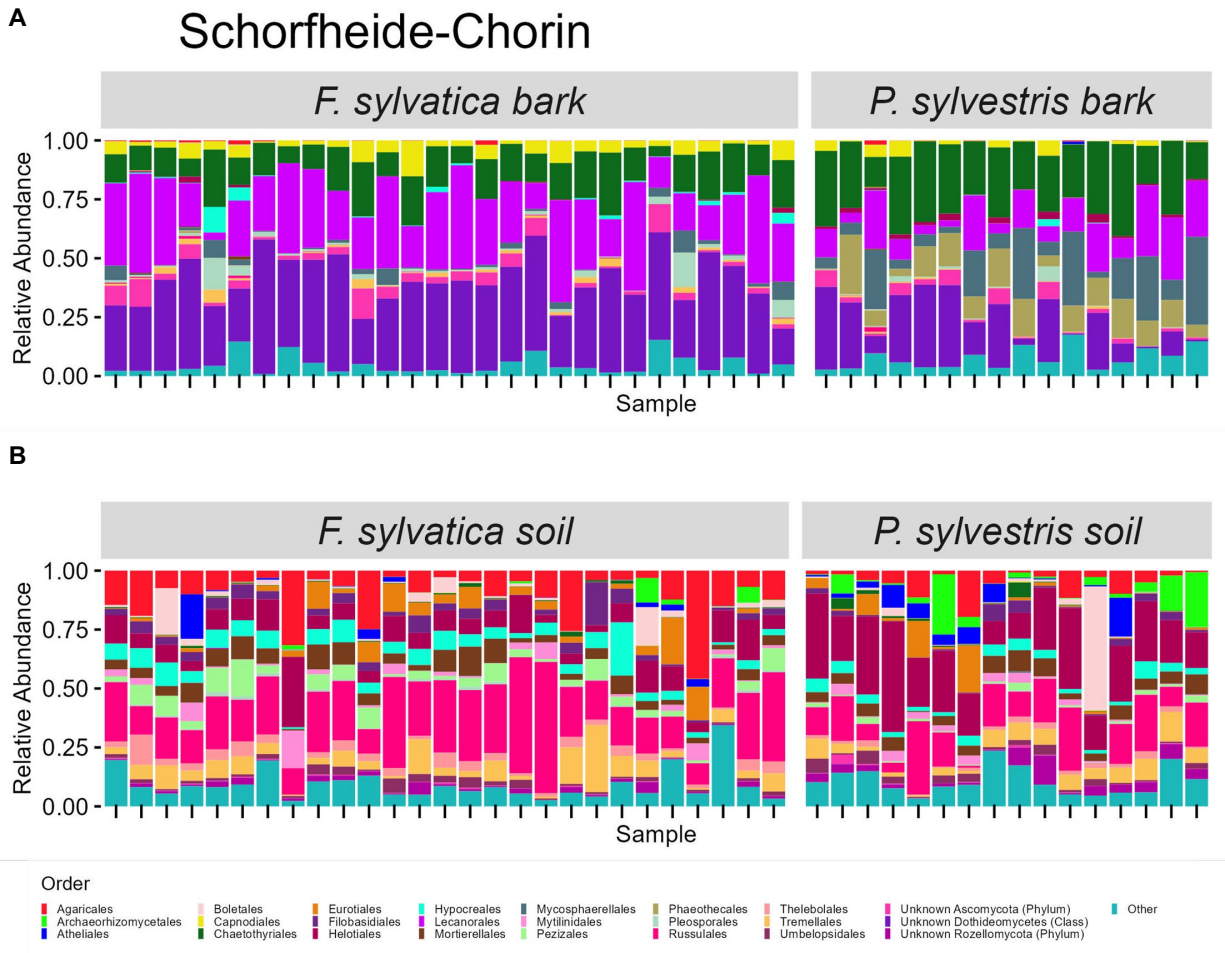
Taxonomic composition of fungal communities in Schorfheide Chorin. **(A)** Fungal groups associated with bark surfaces and **(B)** fungal diversity associated with soil. Taxonomic composition is shown at order level, faceted by dominant tree species. Each bar represents one sample (= one plot) and shows the relative abundances of fungal phyla. Only the 25 most abundant orders per plot are shown. The remaining orders are summarized as ‘other’. Some groups were not assignable at the level of order and are designated with higher taxonomic ranks.

The taxonomic composition of fungal communities at order level differs according to habitat (Figures 4, 5). The most abundant orders in soil (in descending order) are Agaricales, Mortierellales, Russulales in Swabian Alb, and Russulales, Helotiales, Agaricales in Schorfheide-Chorin. On bark surfaces, the most abundant orders, which could be assigned to this taxonomic level, are Lecanorales, Capnodiales, Trapeliales (Swabian Alb), and Lecanorales, Chaetothyriales, Mycosphaerellales (Schorfheide-Chorin). We note, however, that taxonomic assignment yielded ‘unknown Dothideomycetes’ as the second largest group of bark surface communities in both study regions (Figures 4, 5).

Bark surfaces generally contain a high proportion of reads (32–34%) unassignable at the level of order, here designated as ‘unknown Dothideomycetes’, ‘unknown Ascomycota’, ‘unknown Fungi’, and ‘unknown Rozellomycota’. The number of ASVs unassignable to order is 179 (16%) in Swabian Alb, and 114 (15%) in Schorfheide Chorin. Taxonomically unassigned diversity is largely lacking in the soil habitat (Figures 4, 5). Bark surfaces furthermore contain a high proportion of lichenized fungi: 39% of the reads in Swabian Alb, and 23% in Schorfheide Chorin. Swabian Alb features the lichen-forming orders Lecanorales, Trapeliales, Caliciales, and Verrucariales, whereas Schorfheide Chorin mostly contains Lecanorales.

Tree species identity affects the abundance of some orders in soil and on bark surfaces. This effect is different in the two study regions. For example, in bark samples from Swabian Alb the lichen-forming orders Caliciales and Trapeliales are more abundant on the deciduous tree species (*Fagus sylvatica*), whereas the non-lichenized Capnodiales and Mycosphaerellales are more abundant on the coniferous tree species (*Picea abies*; Figure 4A). In bark samples from Schorfheide-Chorin the lichenized Lecanorales are more abundant on the deciduous tree species (*Fagus sylvatica*), whereas Chaetothyriales, Phaeothecales, and Mycosphaerellales are more abundant on the coniferous tree species (*Pinus sylvestris*; Figure 5A). In soil samples from Swabian Alb, Atheliales are more abundant below the coniferous tree species (Figure 4B). In Schorfheide-Chorin, Russulales are more abundant in soil below the deciduous tree species, whereas Archaeorhizomycetales, Helotiales and Atheliales are more abundant in soil below the coniferous tree species (Figure 5B).

### 3.4. Variance partitioning

**Figure 6:**
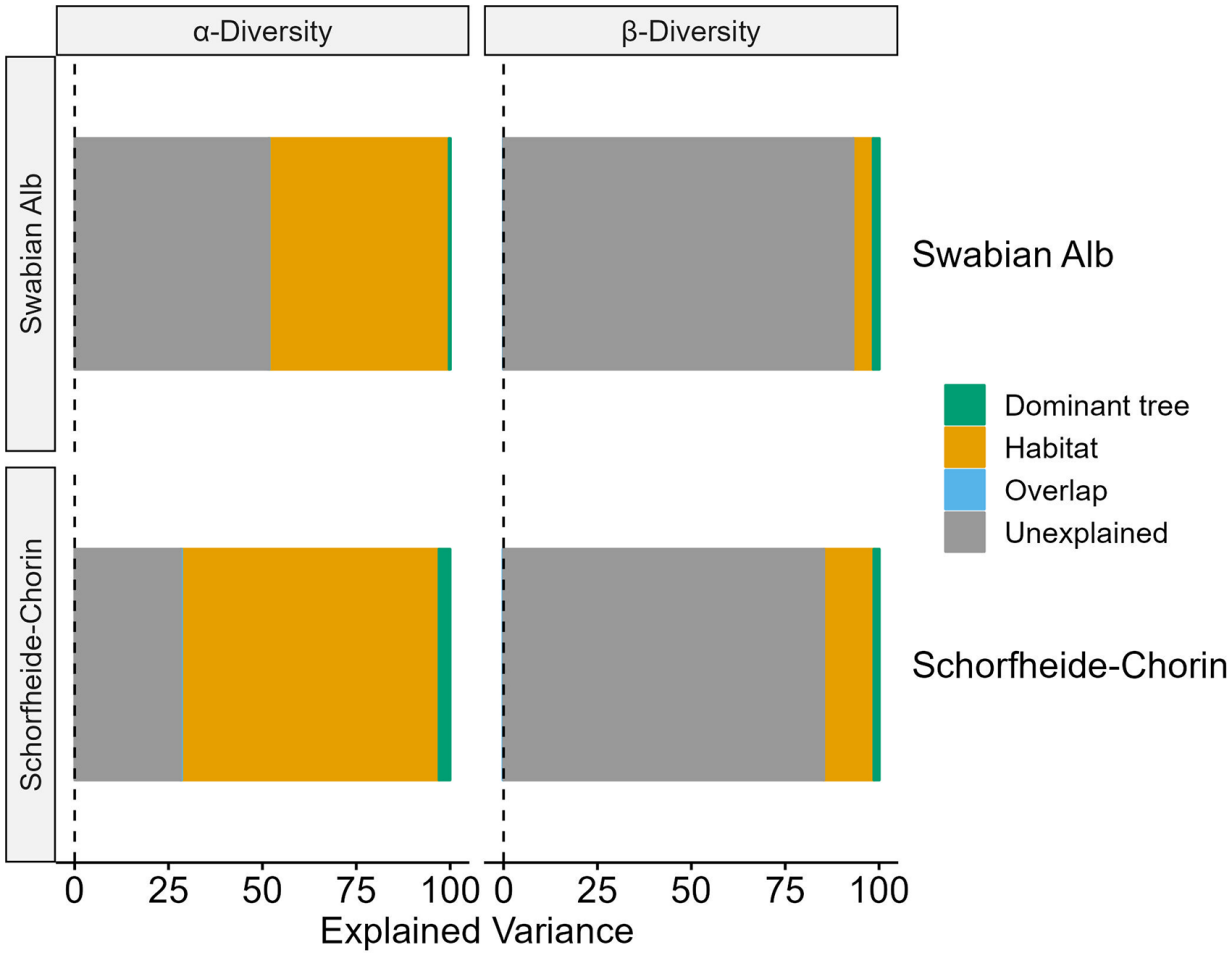
Amount of variation in fungal communities, which can be explained by habitat (bark surface vs. soil), and host tree species in two study regions, Swabian Alb and Schorfheide Chorin. These are results from a variance partitioning analysis. Values are given for alpha and beta diversity.

More than 50% of the variance in alpha diversity can be explained by habitat and tree species, while the majority of variance in beta diversity is unexplained (Figure 6). However, in both cases the habitat explains more variance than the dominant tree species. For the alpha diversity models 48% (Swabian Alb) and 68% (Schorfheide-Chorin) of the variance can be explained by the habitat. Although the overall explained variance is less for beta diversity, we observe a similar allocation between habitat and dominant tree species where habitat explains approximately three times (Swabian Alb) and nine times (Schorfheide-Chorin) more variance than the dominant tree species.

### 3.5. Analysis of co-occurrence networks

**Figure 7:**
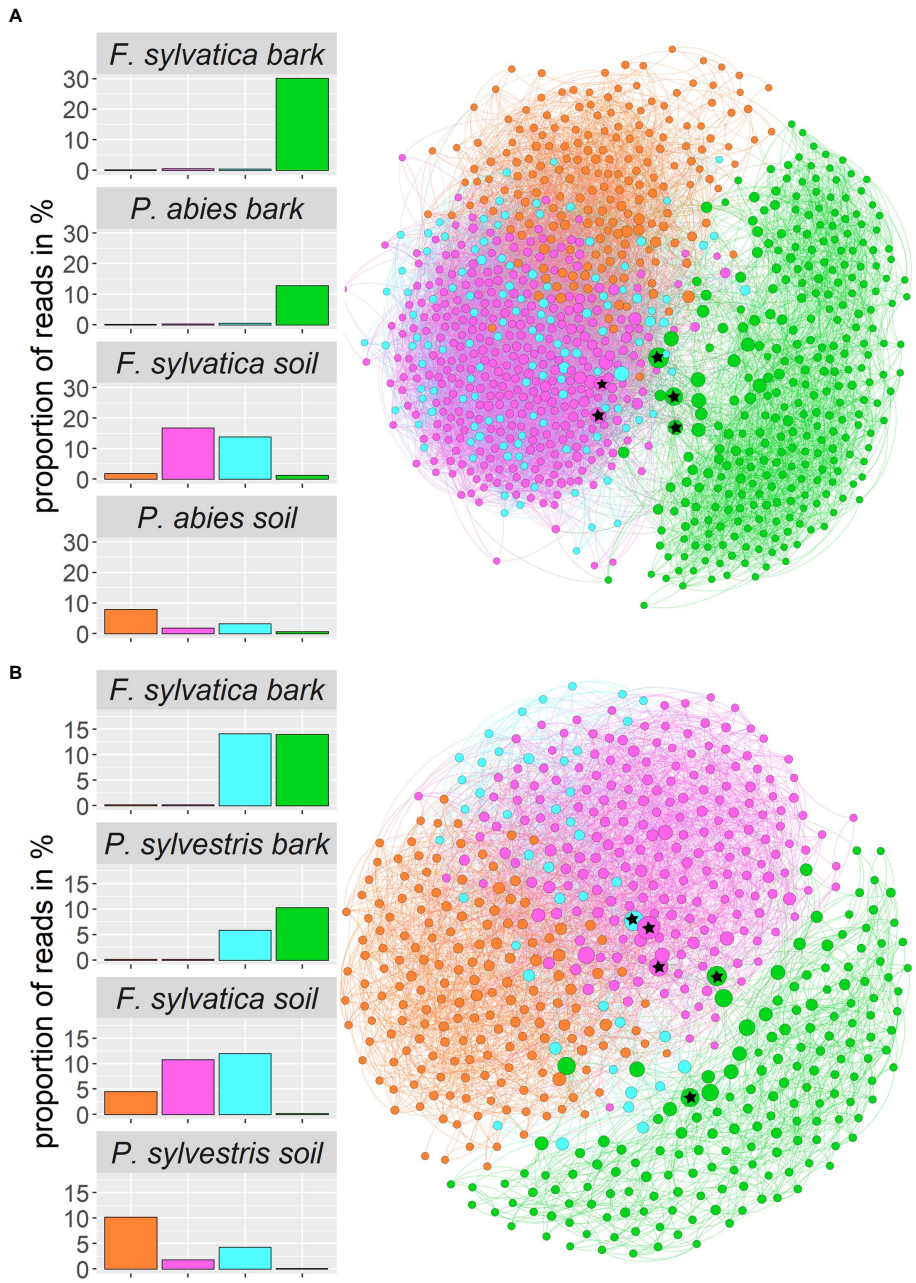
Fungal community structure of the two regions, Swabian Alb **(A)** and Schorfheide-Chorin **(B)**, based on taxon co-occurrence patterns and visualized as networks. Only ASVs with more than 1% relative abundance are included. In both study regions the networks form four subnetworks (modules), which can be allocated to different habitats: Module 0 (pink) contains ASVs specific to the soil of beech-dominated plots, module 1 (green) contains ASVs found on bark, independent of dominant tree species, module 2 (orange) contains ASVs found in soil mostly from coniferous tree species, and module 3 (blue) contains ASVs found on bark and in soil independent of dominant tree species. Bar plots show the proportion of reads within a module and habitat type. The size of the circles indicates the number of connections: larger circles have more connections. The top five hub-taxa for each network are indicated by asterisks in the circles.

We constructed co-occurrence networks for each of the two study regions (Figure 7). Only ASVs that contributed at least 1 % of the total reads entered the network calculations, which resulted in 938 ASVs for the Swabian Alb and 588 ASVs for Schorfheide-Chorin. The network for Swabian Alb is characterized by a diameter of 6 with an average path length of 2.8 and the network for Schorfheide-Chorin by a diameter of 6 and average path length of 3.1. Both networks have a modularity close or higher than 0.4 (Swabian Alb: 0.382; Schorfheide-Chorin: 0.454), indicating a strong division of the networks into modules (subnetworks) (Newman, 2006), and cluster into four modules. One module (green) contains mostly bark-associated fungi and is clearly distinct from the other three modules (Figure 7). The pink module contains mainly ASVs that occur in the soil of *Fagus sylvatica*-dominated plots, while the orange module contains mainly ASVs that occur under soil associated with both coniferous tree species. ASVs in the blue module are found in association with either tree species and either habitat in Schorfheide-Chorin and with soil of both tree species in the Swabian Alb (Figure 7). In general, the networks are similar for both study regions, especially showing a clear distinction between soil and bark.

## 4. Discussion

### 4.1. Geographic location affects fungal diversities, but not overall community patterns

Abiotic factors, such as climatic conditions or soil properties, influence fungal communities associated with trees (e.g., Goldmann et al., 2016; Glassman et al., 2017). Here we study two regions in Germany with different climatic and edaphic conditions and report a strong effect of geographic location on fungal diversities associated with bark and soils. The southern sampling region, Swabian Alb, is significantly more diverse than the northern region, Schorfheide-Chorin (Figure 1). This effect is also visible when considering the same host tree species, *Fagus sylvatica*, in both study regions (Figure 2). Reasons for the lower fungal diversity in the northern region may be lower precipitation and overall humidity, as well as lower nutrient availability, due to sandy soils (Fischer et al., 2010).

The two study regions differ in abiotic conditions, dominant coniferous tree species, and overall fungal diversities. Yet, some general patterns of fungal community composition emerge across all sampling sites: 1. Fungal bark communities are less diverse than soil communities, regardless of host tree species; 2. Forest habitat explains more of the fungal variation than host tree species identity. 3. The number of shared fungal taxa – between habitats and between tree species – is small, but the relative abundance of these taxa is high. 4. Fungal networks indicate a separation of bark and soil communities. Fungal bark communities are less diverse and less known than soil communities.

Forest trees form aboveground and belowground ecological niches that are habitat for diverse fungal communities. It has been suggested that fungi often exploit more than one of these habitats, and serve as connector elements between habitats (Baldrian, 2017), but empirical studies including more than one tree-associated niche are still rare (but see Yang et al., 2022a), and some habitats are poorly known altogether, e.g., the bark surface (Dreyling et al., 2022). Here we contribute to the topic by providing an assessment of fungal communities on bark surfaces of living trees and in soil below these trees, across different tree species in temperate European forests. As a general pattern we observe that bark communities are less diverse than soil communities, regardless of tree species identity. This may be due to lower nutrient availability, and the presence of physical and chemical stressors in bark habitats (Buck et al., 1998; Baldrian, 2017). In contrast, soil contains ample carbon and nutrient sources, as well as various micro niches, supporting diverse fungal communities (Baldrian, 2017).

A striking result of this work is the high proportion of bark-associated fungal reads, which are not assignable at the level of order. This is consistent with an earlier study in the Hainich-Dün Exploratory in central Germany, which included only beech forests (Dreyling et al., 2022). In the present study we report that approximately 16% of the ASVs, corresponding to 33% relative abundance, on bark, cannot be assigned at order level. The fraction of “unknown Dothideomycetes” is particularly prominent across bark surfaces of all tree species in all study regions (Figures 4, 5). In contrast, less than 5% (relative abundance) of the fungi found in soil are unassignable at order level. These results, found in both study regions, and across all tree species, highlight gaps in our knowledge of bark-associated fungal communities in temperate forests. Bien and Damm (2020) found a considerable number of fungi that are not assignable at species or genus level in the wood of fruit trees. Other studies of fungal bark communities focused so far on certain groups, like yeasts (Bhadra et al., 2008), lichens (Aschenbrenner et al., 2017), or endophytic fungi (Pellitier et al., 2019), and did not consider the entire community of fungi that occur on the substrate. The undescribed fungal diversity on tree bark underlines our poor knowledge of aboveground plant-microbiome relationships (e.g., Aschenbrenner et al., 2017), and fungal diversity in general, with less than an estimated 10% of fungi scientifically described (Hawksworth and Lücking, 2017).

Community composition differs between bark and soil communities: Bark is dominated by ascomycete orders, whereas soil is dominated by basidiomycete orders. The dominance of Basidiomycota in forest soils (including ectomycorrhizal fungi) has been known since the earliest metabarcoding studies (e.g., Buée et al., 2009). The dominance of Ascomycota on bark was shown for apple and pear trees by Arrigoni et al. (2018).

Tree bark is a typical habitat for lichenized fungi, and as expected, a large proportion of bark-associated reads belong to lichen-forming fungal orders. Tree species identity drives part of the lichen flora in Swabian Alb, where Caliciales and Trapeliales are much more prominent on beech than on spruce, adding to the higher overall diversity of lichens on beech. This is in line with a floristic study, which showed that deciduous forests in Swabian Alb are more diverse than coniferous forests with respect to lichenized fungi (Boch et al., 2013). Most tree trunks sampled in the present study were not visibly covered with lichens directly at the sampling sites. However, we likely collected lichen propagules like soredia, thallus fragments, or spores, which adhered to the bark at the sampling site, and which may have been transported there by stemflow or wind (Magyar et al., 2021). The presence of lichens may be connected to the large amount of unassigned fungal reads on bark surfaces: The lichens themselves constitute an ecological niche associated with a particularly high proportion of unknown fungal species, compared to other fungal habitats worldwide (Baldrian et al., 2022).

### 4.2. Forest habitat explains more of the fungal variation than host tree species identity

Aboveground and belowground habitats provided by trees are characterized by different fungal communities (e.g., Durand et al., 2017). Here we confirm that much of the variance in alpha diversity is explained by habitat, however, a small portion is explained by tree species identity (Figure 6). The number of fungal taxa shared between aboveground and belowground habitats is low, but the abundance of these taxa is high.

Tree species identity has been described as an important driver of fungal community composition in some forest habitats, such as litter (Prescott and Grayston, 2013; Urbanová et al., 2015). In the present study the bulk of the fungal community did not respond to tree species identity. Different tree species share more than 75% of the fungal reads in the soil habitat, and more than 80% in the bark habitat (Figure 2). However, in both sampling regions, a small portion (less than 30%) of fungal reads was found either on the coniferous or the deciduous tree species, in both habitats, bark, and soil (Figure 2B).

Bark traits of the host trees may account for differences of the associated fungal communities. They include physical texture, water storage capacity, mineral content, pH, chemical composition, and stability. The bark surfaces of most *Fagus sylvatica* trees sampled in this study were smooth and unbroken, but older trees occasionally featured deep crevices. Bark surfaces of *Picea abies* were rough and scaly with many cracks, while those of *Pinus sylvestris* consisted of flaking corky ridges, separated by vertical furrows. The bark of *Picea abies* and *Pinus sylvestris* is slightly more acidic (pH 4–5) (Prasetya and Roffael, 1990) than the bark of *Fagus sylvatica* (pH 4.9–7.0) (Fritz and Heilmann-Clausen, 2010). Furthermore, beech bark contains phenolic substances with antibiotic properties (Tănase et al., 2018).

Differences in light availability at the tree trunks in coniferous versus deciduous forests may influence the local community of light-dependent fungi, such as lichenized fungi. Tree species identity and bark traits are known to influence fungal communities on decaying bark and wood (Yang et al., 2022b). It is presently not known whether fungal communities of living bark affect the communities that assemble on dead bark, e.g., through priority effects, or by switching to a saprotrophic lifestyle (Rai and Agarkar, 2016).

A previous study in the Biodiversity Exploratories found that the fungal soil community differed significantly between beech-dominated and coniferous tree-dominated stands (Goldmann et al., 2015). In the present study the tree species effect is slightly more visible in fungal communities in soil than on bark. Possible reasons for this could be the presence of specialized fungal degraders associated with different litter types (Barbi et al., 2016), or the presence of tree species-specific ectomycorrhizal communities (Goldmann et al., 2015; Nacke et al., 2016).

### 4.3. Ecological networks indicate largely separated above- and belowground fungal communities, with some connecting elements

Aboveground-belowground species interactions affect ecosystem properties, especially at local scales (Deyn and Van Der Putten Wim, 2005). In the forest ecosystem, fungi have been suggested to be connecting elements between different habitats (Baldrian, 2017), yet we know little about the interaction of above- and belowground fungal communities in temperate forests. Fungal communities associated with different tree compartments differ, as it has been shown for poplar trees (Durand et al., 2017), and phyllosphere versus root and soil fungi associated with forest trees in subtropical forests (Yang et al., 2022a). In the present study we use co-occurrence networks to better understand the differences and linkages between aboveground (bark surface associated) and belowground (soil associated) fungal communities. Networks comprising all fungi of one study region are strikingly similar for the northern and southern study region. One module (containing bark-associated fungi) is clearly distinct from the other three modules. Another module (blue) contains ASVs that are found in samples from both habitats and tree species. We hypothesize that these fungi are able to connect the different habitats. One potential pathway for the connection of the aboveground- and belowground habitats is stemflow (Magyar et al., 2021). Rainwater, which runs down the branches and stems of trees carries a multitude of microorganisms, which are washed into the ground, and may become part of the soil microbiome (van Stan et al., 2020; Magyar et al., 2021; Teachey et al., 2022). Conversely, fungal particles, such as spores, could be transported by wind in the other direction, from soil to bark, and adhere to bark surfaces and bark biofilms. While the bark community forms a single module including fungi from all host tree species, the soil community differentiates into more than one module: one module contains soil fungi in general, another module contains predominantly soil fungi from beech stands. Boraks et al. (2021) describe a similar pattern for tropical fungi, and suggest that belowground fungal communities respond to tree species composition, while aboveground community turnover is more dependent on geographical distances.

So-called hub taxa are important “connectors” that ensure stability and functioning of the network. These taxa have the highest betweenness centrality, i.e., the highest number of shortest paths going through them. ASV 28 (‘*Phallus impudicus*’) and ASV_257 (‘*Cladosporium’*) are identified as hub taxa in both study regions. *Phallus impudicus*, the common stinkhorn, occurs in soil and – less abundantly – on bark surfaces. It is a widespread saprotrophic mushroom usually found in soil. Dispersal occurs *via* insects, which may explain a wide distribution of environmental DNA of this fungus also in aboveground habitats. The genus *Cladosporium* is cosmopolitan and occupies virtually all ecological niches (Bensch et al., 2012). It is regularly detected in aerobiological samples, also using eDNA methods (Tordoni et al., 2021). Thus, ASV_257 (‘*Cladosporium’*) appears to be a widespread member of this genus. One of the hub taxa identified in Schorfheide-Chorin is *Phylctis argena*, a common crustose lichen on bark, which we frequently encountered in all of the Biodiversity Exploratories. It forms thin thalli on bark, mostly on deciduous trees. Tiny dispersal propagules (soredia) cover individuals of this lichenized fungus and likely account for the wide aboveground and belowground distribution, as they can be transported to other trees by wind, and into the soil by stemflow. *Neocucurbitaria quercina* is one of the hub taxa identified in Swabian Alb. It belongs to a family of plant-associated or plant pathogenic fungi (Hyde et al., 2013; Wanasinghe et al., 2017). The other hub taxa are taxonomically not assignable at species level, underlining our incomplete knowledge of forest biodiversity also with regard to species with potential importance at ecosystem level.

## 5. Caveats and conclusion

Ecological inferences based on metabarcoding data are sensitive to the way the amplicons are treated, and it is an ongoing debate whether amplicon sequencing variants (ASVs), or operational taxonomic units (OTUs) better represent fungal communities. Here, we opted for ASVs, because they provide higher accuracy over 97% identity OTUs. Nonetheless, we are aware of limitations of the ASV approach, such as the risk of splitting species into separate ASVs, because of different rRNA copies present in the same genome (Schloss, 2021). Recent comparative studies drew opposing conclusions as to which method to use, either favoring ASVs (e.g., Joos et al., 2020; Cholet et al., 2022), or OTUs (Tedersoo et al., 2022). We argue that to date, there is no consensus for the most appropriate method for amplicon treatment, and for the time being, both approaches are acceptable.

Overall, our study contributes knowledge to fungal diversity patterns in temperate forests, and the connections between aboveground and belowground fungal habitats. We show that rather few, but highly abundant fungal taxa overlap between habitats and tree species. It remains to be seen, if these taxa are random environmental fungi, or if they are part of a core “forest mycobiome.” Our study also provides baseline data of fungi associated with common European tree species, which are potentially useful for biogeographic studies, or biodiversity monitoring. We show that lichenized fungi can be detected in eDNA swabbed from bark surfaces. These data could be assessed for their use in future forest lichen inventories. We also confirm gaps in our knowledge of fungal communities associated with bark surfaces. Future studies of this type, integrating over additional forest habitats, and more organismal groups, will potentially unravel not only diversity patterns and interactions, but also vulnerabilities of the forest ecosystem.

## Data availability statement

The raw sequences are deposited in the NCBI SRA repository, accession number SRR23371988. All scripts and additional data necessary to replicate the analysis are available at https://github.com/LukDrey/fungal_habitat. The selection of the dominant tree species is based on a stand composition assessment available at https://www.bexis.uni-jena.de/ under Accession number 22907.

## Author contributions

IS, FG, and LD conceived and planned the study. LD, IS, FG, and BH collected the samples. LD and JO generated the molecular data. BH and LD analyzed the data. BH, IS, and LD wrote the manuscript with contributions from all authors. All authors contributed to the article and approved the submitted version.

## Funding

This work has been partly funded by the DFG Priority Program 1374 “Biodiversity-Exploratories” (SCHM 1711/8-1). Field work permits were issued by the responsible state environmental offices of Baden-Württemberg and Brandenburg.

## Conflict of interest

The authors declare that the research was conducted in the absence of any commercial or financial relationships that could be construed as a potential conflict of interest.

## Publisher’s note

All claims expressed in this article are solely those of the authors and do not necessarily represent those of their affiliated organizations, or those of the publisher, the editors and the reviewers. Any product that may be evaluated in this article, or claim that may be made by its manufacturer, is not guaranteed or endorsed by the publisher.

## Footnotes

^1^ http://www.biodiversity-exploratories.de/en/
