# Supplementary figures and images for: Habitat and tree species identity shape aboveground and belowground fungal communities in central European forests
Source: Front Microbiol. 2023 Mar 6;14:1067906. doi: 10.3389/fmicb.2023.1067906 (PMC10025312; doi:10.3389/fmicb.2023.1067906)

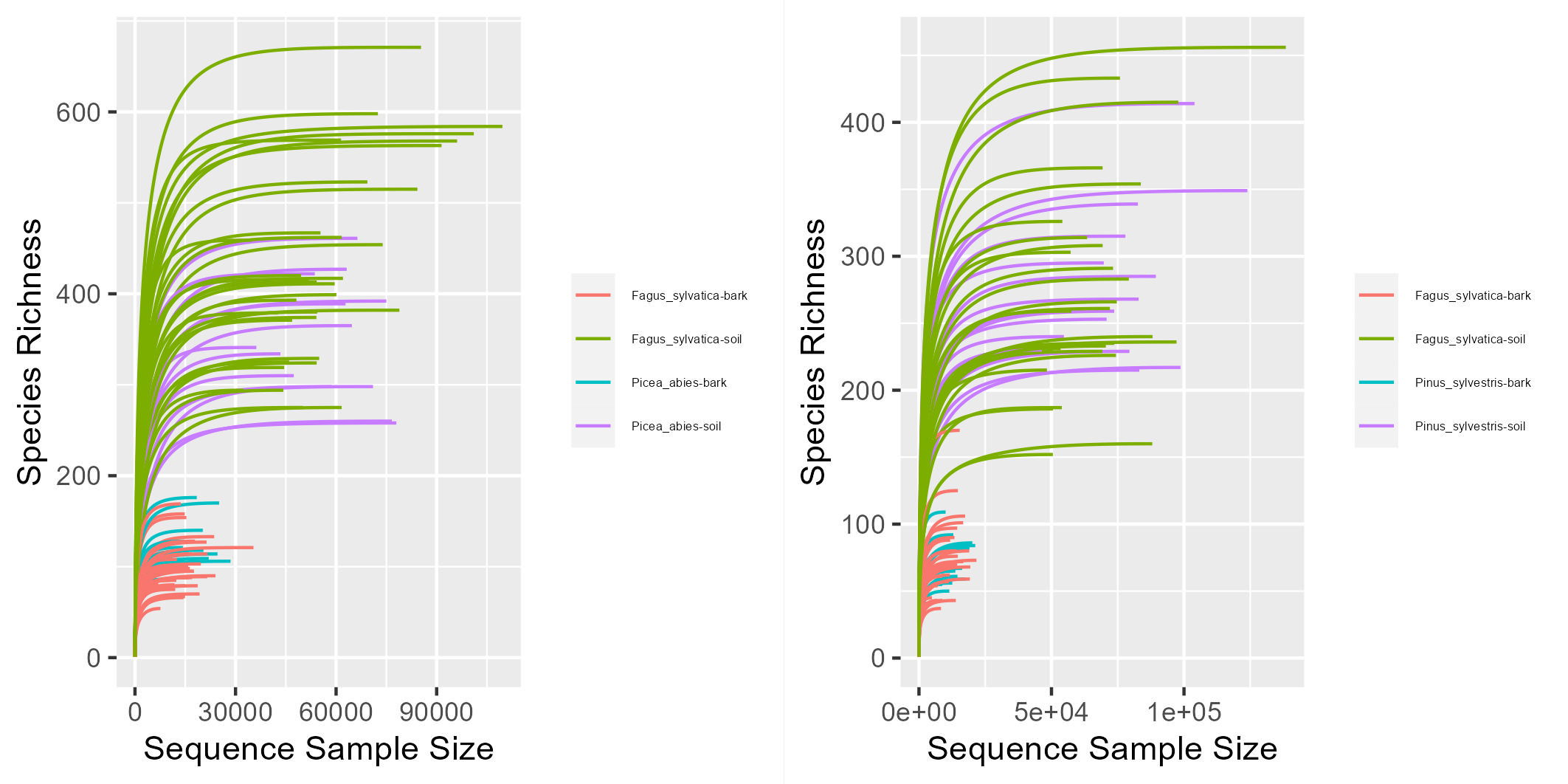

Supplement: Supplementary file 1 [file Image_1.JPEG]

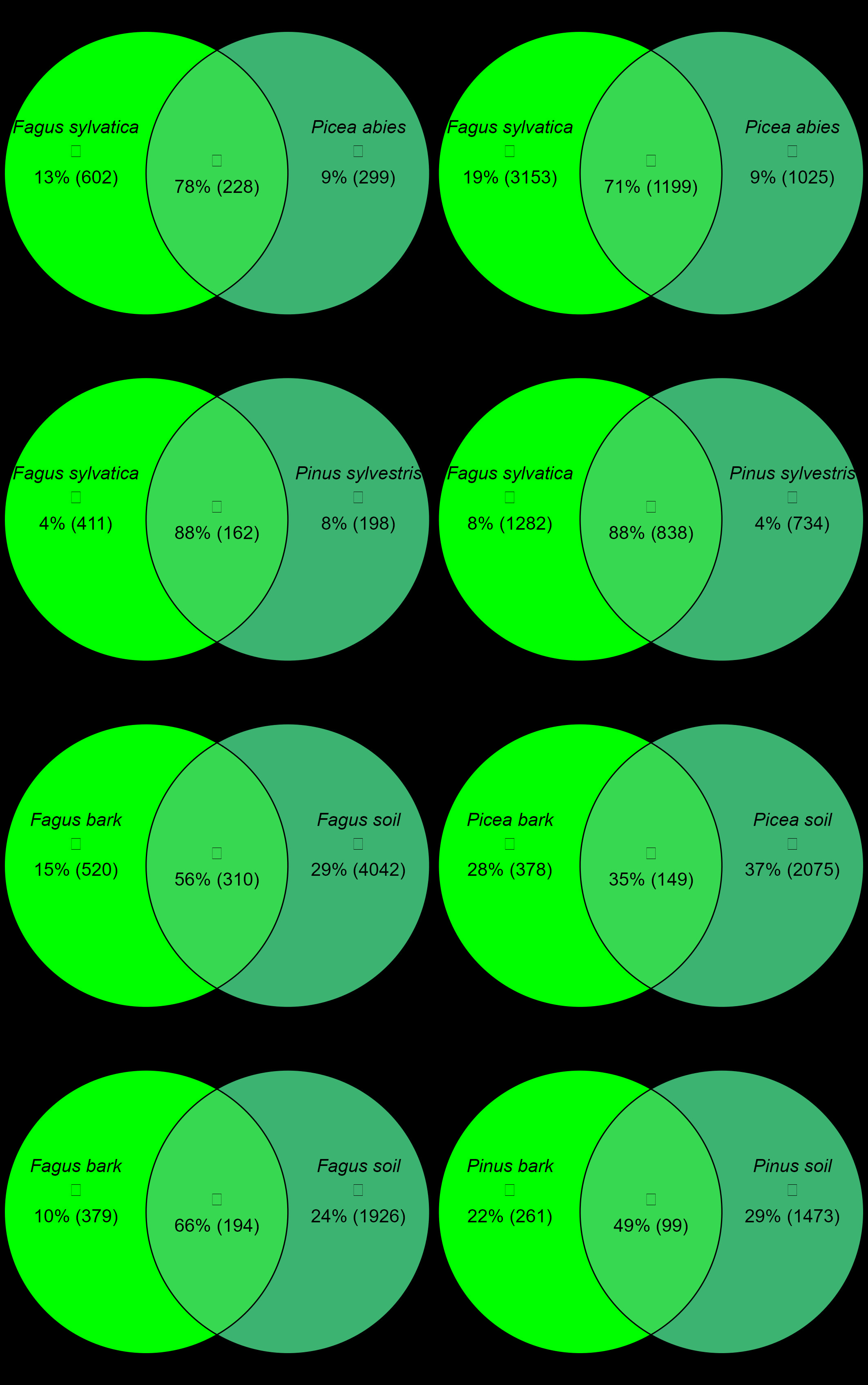

Supplement: Supplementary file 2 [file Image_2.JPEG]
